# DRug-coated Balloon for Endovascular treatment of sYmptOmatic intracraNial stenotic Disease (DR. BEYOND): the protocol of a multicentre randomised trial

**DOI:** 10.1136/svn-2024-003259

**Published:** 2024-07-23

**Authors:** Dapeng Mo, Xu Tong, Xiaoqing Li, Chuan Qin, Yuesong Pan, Sheng Guan, Zhongrong Miao

**Affiliations:** 1 Interventional Neuroradiology, Department of Neurology, Beijing Tiantan Hospital, Capital Medical University, Beijing, China; 2 Department of Quality, Beijing Taijieweiye Technology Co., Ltd, Beijing, China; 3 China National Clinical Research Center for Neurological Diseases, Beijing Tiantan Hospital, Capital Medical University, Beijing, China; 4 Department of Neurointervention, The First Affiliated Hospital of Zhengzhou University, Zhengzhou, China

**Keywords:** Angioplasty, Atherosclerosis, Clinical Trial, Intervention, Ischemic Stroke

## Abstract

**Background:**

Although endovascular stenting is considered an effective and safe therapeutic option for symptomatic intracranial atherosclerotic disease (sICAD), an elevated rate of restenosis remains an important issue for the conventional bare-metal stent (BMS). Recent evidence from observational studies suggests that applying drug-coated balloons (DCB) in sICAD may decrease restenosis occurrence. Additional large randomised studies are warranted to provide firmer evidence and to determine which patients would benefit most from DCB.

**Aim:**

To design a randomised trial to examine DCB angioplasty (Taijieweiye intracranial paclitaxel-coated balloon catheter) versus BMS stenting (Wingspan intracranial stent system) in patients with sICAD.

**Design:**

This is a multicentre, prospective, randomised, open-label, blinded end-point study to assess whether DCB angioplasty reduces the risk of restenosis compared with BMS stenting in sICAD patients with high-grade stenosis (≥70%–99%). Our goal is to randomly assign 198 eligible individuals at a 1:1 ratio to undergo DCB angioplasty (intervention group) or BMS stenting (control group).

**Outcome:**

The primary efficacy outcome is restenosis at 6 months post treatment, that is, >50% stenosis in or within 5 mm of the treated segment and >20% absolute luminal loss. The primary safety outcome is stroke or death within 30 days post treatment.

**Discussion:**

The DRug-coated Balloon for Endovascular treatment of sYmptOmatic intracraNial stenotic Disease trial aims to produce strong evidence on the efficacy and safety of DCB angioplasty as a promising therapeutic option for sICAD cases with high-grade stenosis.

**WHAT IS ALREADY KNOWN ON THIS TOPIC**

- Several observational studies have reported promising findings about drug-coated balloons (DCB) angioplasty in symptomatic intracranial atherosclerotic disease (sICAD) patients, with low restenosis and complication rates. The next logical step is that randomised studies may inform whether DCB angioplasty is non-inferior or even superior to conventional bare-metal stents (BMS) stenting.

**WHAT THIS STUDY ADDS**

- This protocol provides the rationale and design of DRug-coated Balloon for Endovascular treatment of sYmptOmatic intracraNial stenotic Disease.

**HOW THIS STUDY MIGHT AFFECT RESEARCH, PRACTICE OR POLICY**

- The present study will generate objective data assessing the efficacy and safety of DCB angioplasty versus BMS stenting in sICAD patients with high-grade stenosis.

## Introduction

 Symptomatic intracranial atherosclerotic disease (sICAD) represents an important pathology in the cerebrovascular field.^1^ Current guidelines recommend best medical treatment (BMT), which combines antiplatelet treatment and vascular risk factor control, as the first-line treatment, while endovascular treatment (EVT) is applied as a rescue therapy.^2^ Even after BMT, a high rate of stroke recurrence is detected in sICAD cases, particularly in individuals with high-grade stenosis.^3^ In addition, three randomised studies with large samples (Stenting and Aggressive Medical Management for Preventing Recurrent Stroke in Intracranial Stenosis (SAMMPRIS), Vitesse Intracranial Stent Study for Ischemic Stroke Therapy (VISSIT) and China Angioplasty and Stenting for Symptomatic Intracranial Severe Stenosis (CASSISS)) could not prove the superiority of stenting using bare-metal stents (BMS) over BMT in patients with sICAD due to the high incidence rates of restenosis and other periprocedural complications.^3^,^5^ The restenosis rate may reach 30% for BMS, which is a long-term sequela accounting for one-third of recurrent ischaemic events.^6 7^ Restenosis mostly results from neointimal hyperplasia, so drug-eluted stents (DES) and drug-coated balloons (DCB) were developed to address this problem, and mitotic inhibitors (eg, paclitaxel) or immunomodulators (eg, sirolimus) are frequently used for DES and DCB coating.^8^ A recent study showed that DES can reduce the risks of restenosis and recurrence of ischaemic stroke in sICAD patients with high-grade stenosis.^9^ Currently, there are no comparable studies of DCB angioplasty and DES stenting for sICAD. Theoretically, DCB angioplasty offers several advantages over BMS or DES stenting, including no residual foreign bodies and uniformly distributed drug coverage in the vascular lumen, positive remodelling and reduced dual antiplatelet therapy (DAPT) procedure time.^10^ DCB angioplasty is often applied for interventional cardiology with good safety and efficacy profiles.^11^ Correspondingly, DCB might be promising for EVT in clinical sICAD.

Two retrospective comparative trials in sICAD cases have reported that DCB angioplasty offers several advantages over BMS stenting, suggesting DCB as a promising therapeutic tool for sICAD.^12 13^ However, randomised controlled trials (RCTs) to further support the results of observational studies are lacking. Here, we designed the ‘DRug-coated Balloon for Endovascular treatment of sYmptOmatic intracraNial stenotic Disease (DR. BEYOND)’ RCT to examine whether DCB angioplasty is non-inferior or even superior to BMS stenting in sICAD cases with high-grade stenosis.

## Methods

### Design and study population

This multicentre, prospective, randomised, open-label, blinded end-point study is designed to examine the efficacy and safety of DCB angioplasty for sICAD treatment. In total, 198 eligible patients are planned to be recruited in 14 comprehensive stroke centres across China. Box 1 summarises the inclusion and exclusion criteria.

Box 1 **Eligibility criteria**

**Inclusion criteria:**

1. Age 18–80 years.
2. Symptomatic intracranial atherosclerotic disease (sICAD) patients with recurrent or progressive stroke/transient ischaemic attack within the past 180 days despite best medical treatment. All cases were under at least one antiplatelet agent or oral anticoagulant, receiving high-dose statins. Additionally, lifestyle change and/or drug administration was performed for risk factor control of secondary stroke.
3. sICAD with high-grade stenosis (≥70%–99%) determined by the warfarin–aspirin symptomatic intracranial disease^14^ technique using digital subtraction angiography with ≤15 mm lesion length and ≥2 mm diameter in the target intracranial vessels, including distal segment of the internal carotid artery, M1 segment of the middle cerebral artery, V4 segment of the vertebral artery and the basilar artery.
4. sICAD caused by hypoperfusion with poor collaterals was assessed by three criteria,^23^ including (1) American Society of Interventional and Therapeutic Neuroradiology/Society of Interventional Radiology Score<3,^24^ (2) decrease of >30% in cerebral blood flow in the territory distal to the target lesion by CT or MR perfusion and (3) haemodynamic ischaemic lesions diagnosed by MRI or CT.
5. All patients or their authorised family members provided signed informed consent prior to enrolment.

**Exclusion criteria:**

1. Anatomic factors such as severe calcification or distortion of the target vessel make it difficult for the study device to reach the lesion site.
2. Severe dissection with limited blood flow or excessive residual stenosis resulting from lesion calcification or elastic recoil after predilation with conventional balloon(s).
3. Severe stenosis or total occlusion of tandem extracranial or intracranial vessels found proximal or distal to the target vessel.
4. Acute ischaemic stroke or major surgery in the past 3 weeks.
5. Cerebral haemorrhage, massive cerebral infarction, cardiogenic stroke, myocardial infarction within the past 30 days.
6. A history of stenting for the target lesion.
7. Non-atherosclerotic stenosis caused by arterial dissection, moyamoya disease, vasculitis, radiation angiopathy or fibromuscular dysplasia.
8. Preenrolment modified Rankin Scale Score≥3.
9. Coagulation dysfunction or irreversible bleeding.
10. Inability to tolerate general anaesthesia, contraindication or severe allergy to procedure-related drugs (paclitaxel, heparin, contrast agent, aspirin or clopidogrel, etc) or study devices.
11. Platelet count<90×10^9^ /L, haematocrit<30%, international normalised ratio (INR)>1.5, severe hypertension refractory to medication (systolic blood pressure>180 mm Hg or diastolic blood pressure>110 mm Hg), severe heart or lung failure, severe liver impairment (alanine transaminase (ALT) or aspartate transaminase (AST)>3 times the upper limit of normal) or kidney dysfunction (serum creatinine>3 mg/dL).
12. Current participation in other studies applying drugs or devices.
13. Pregnancy or lactation in women, or pregnancy planning within 1 year.
14. Inability to follow-up because of cognitive or emotional diseases or mental disease.
15. Life expectancy<3 year.
16. Other conditions rendering the patient unsuitable for enrolment per investigators’ judgement.

### Randomisation

**Figure 1:**
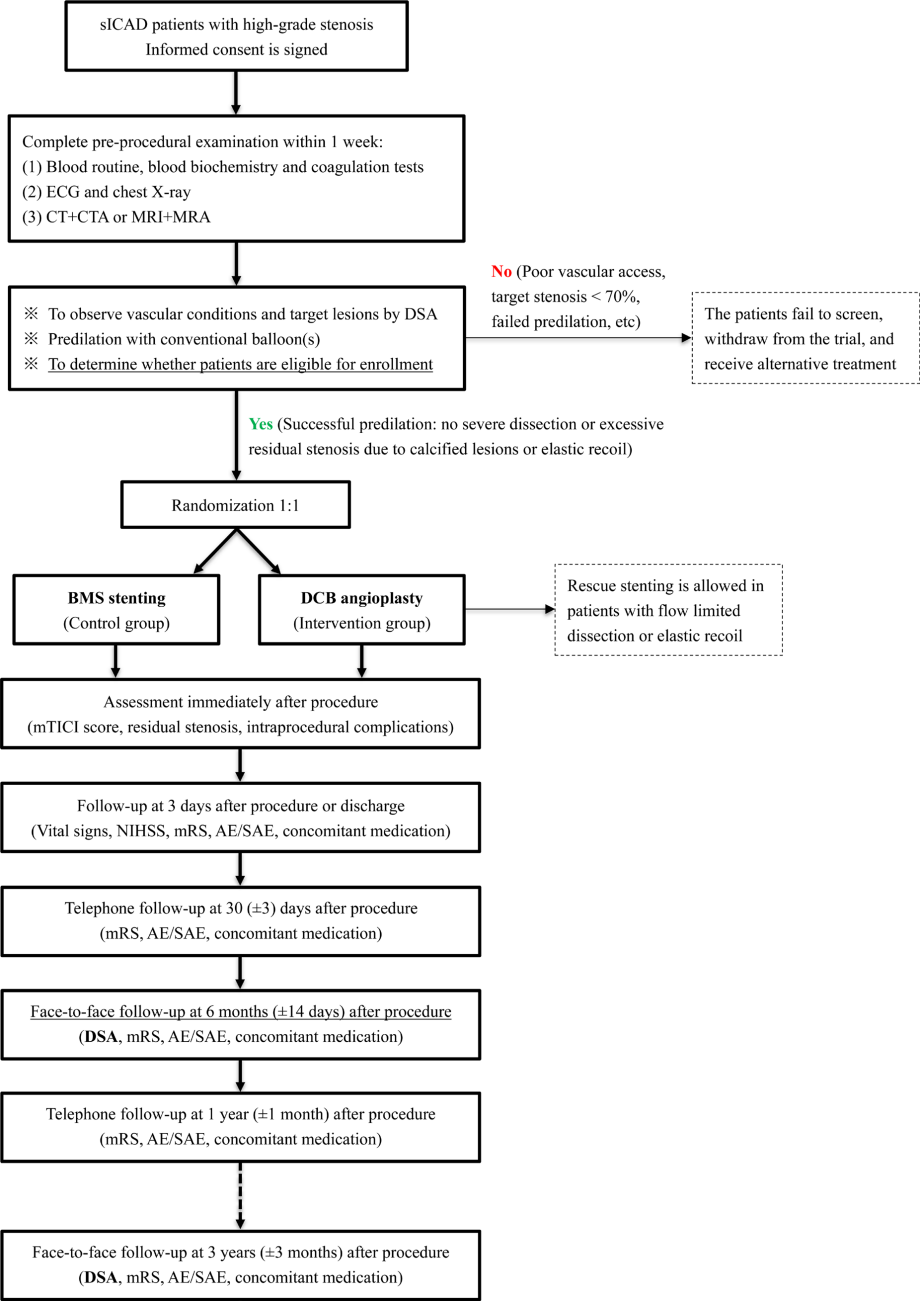
The flowchart of DR. BEYOND trial. AE, adverse event; BMS, bare-metal stent; CTA, CT angiography; CTP, CT perfusion; DCB, drug-coated balloon; DR. BEYOND, DRug-coated Balloon for Endovascular treatment of sYmptOmatic intracraNial stenotic Disease; DSA, digital subtraction angiography; MRA, MR angiography; mRS, modified Rankin Scale; mTICI, modified Thrombolysis In Cerebral Infarction; NIHSS, National Institutes of Health Stroke Scale; PWI, perfusion weighted imaging; SAE, serious adverse event; sICAD, symptomatic intracranial atherosclerotic disease.

Eligible individuals will be randomised into two groups (DCB angioplasty and BMS stenting) at a 1:1 ratio using the block randomisation method stratified by study centre (figure 1). The table of random numbers generated through computer software is placed in a sealed and opaque envelope (allocation concealment) and is managed and distributed by a designated person in the main study centre (located at Beijing Tiantan Hospital).

### Intervention

#### Pre procedure

DAPT with aspirin (100 mg/day) and clopidogrel (75 mg/day) or ticagrelor (180 mg/day) is routinely maintained for at least 5 days prior to the procedure. Thromboelastography and/or CYP2C19 genotype testing are recommended to assess the occurrence of clopidogrel resistance, and clopidogrel is switched to ticagrelor in patients with clopidogrel resistance. Endovascular procedures are carried out under general anaesthesia using a 6F or 8F guiding catheter through the right femoral artery. Bolus intravenous heparin is administered to achieve an activated clotting time of 250–300 s for the procedure. The stenosis degree is determined according to the warfarin–aspirin symptomatic intracranial disease study.^14^ After conventional cerebral angiography, two device systems are applied for intervention, including a paclitaxel-coated balloon (Taijieweiye, China) particularly developed for neurovascular application (length and diameter of 9–30 and 1.5–4.0 mm, respectively), and a Wingspan stent (Stryker Neurovascular, USA) approved by National Medical Products Administration for intracranial use as the reference stenting system. The vessel pathway is fully assessed before the procedure, applying a distal access catheter in case of highly tortuous vessel pathway.

#### DCB angioplasty (intervention group)

**Figure 2:**
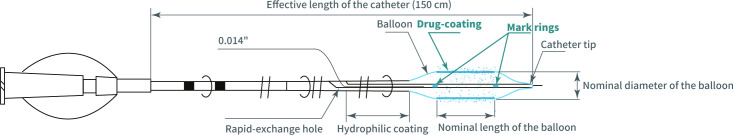
Device diagram.

The device used in the intervention group is a rapid-exchange DCB catheter system comprising six parts, that is, a DCB, marker ring, distal and proximal rods, guidewire channel and catheter socket (figure 2). The balloon at the distal rod’s end might be expanded to a given diameter under the recommended pressure. Each balloon has two marker rings on its two shoulders for visualisation and localisation during the procedure. Paclitaxel (1.5 μg/mm^2^) is used to coat the balloon. The guidewire channel in the distal rod’s lumen accommodates a guidewire (diameter≤0.014″).

Under roadmap guidance, a 0.014″ guidewire crosses the stenosis. The guidewire’s tip is placed distally to the lesion. Each lesion is first predilated with conventional balloons that facilitate the advancement of DCB (higher rigidity) in the lesion. In case of no severe dissection or pronounced residual stenosis resulting from lesion calcification or elastic recoil after predilation with conventional balloon(s), further angioplasty employing a DCB is performed right away. The DCB is chosen with a diameter corresponding to approximately 60%–80% of the normal vessel’s diameter and the same diameter as or 0.25–0.5 mm larger compared with the conventional balloon. The DCB catheter is navigated via the guidewire to cover the whole lesion and to exceed the lesion edge by 1–3 mm. The DCB undergoes a slow inflation to achieve the nominal pressure and remains inflated for 60 s. Angiography is carried out right after DCB deflation and 15–20 min thereafter to prevent any worsening dissection, thrombus or arterial recoil. Rescue stenting is allowed, except the use of Wingspan stent, in patients with flow-limited dissection or elastic recoil. In individuals with thrombus or mild dissection, tirofiban is administered intravenously with an initial dose of 0.4 µg/kg/min for half an hour and a maintenance dose of 0.1 µg/kg/min for 24 hours.

#### BMS stenting (control group)

The Wingspan stent is directly implanted by the over-the-wire technique after adequate predilation with conventional balloon(s). Several studies have described the procedure of the Wingspan stent for treating sICAD.^3 5 15^

#### Post procedure

Other procedure-related complications are resolved per the operator’s discretion. Postprocedurally, Dyna-CT is carried out to exclude any bleeding. The patients are maintained under DAPT using aspirin and clopidogrel or ticagrelor for 3 months. Additionally, all patients receive statin therapy, and vascular risk factors (eg, hypertension and diabetes) are controlled by administration of adequate drugs.

#### Data collection and follow-up outcomes

Clinicodemographic, angiographic and periprocedural indexes will be recorded. Cases are followed up at 3 days (or discharge), 30 (±3) days, 6 months (±14 days), 1 year (±1 month) and 3 years (±3 months). They are scheduled to return for a digital subtraction angiography (DSA) examination at 6 (±1) months and 3 years (±3 months) after the index procedure. Online supplemental table S1 lists the detailed assessment and follow-up schedule. The imaging and clinical outcomes are reviewed by the members of imaging core-laboratory (Core-Lab) and clinical events committee (CEC), separately, who are blinded to the treatment allocation. Disagreements are resolved by consensus.

#### Primary efficacy outcome

Restenosis at 6 months, which is reflected by >50% stenosis in or within 5 mm of the treated segment and >20% absolute luminal loss assessed by DSA.^13^

#### Secondary efficacy outcomes

1. Technical success, which is reflected by <50% residual stenosis postprocedurally.
2. Symptomatic restenosis at 6 months, which is reflected by restenosis related to ischaemic symptoms of the offending vessel’s territory.
3. Recurrent ischaemic events (stroke or transient ischaemic attack (TIA)) beyond 30 days through 1 year.
4. Modified Rankin Scale (mRS) Score at 1 year.

#### Primary safety outcome

Stroke or death within 30 days.

#### Secondary safety outcomes

1. Procedure-related complications (arterial dissection, arterial perforation, vasospasm requiring treatment, distal embolization, thrombus formation, etc)
2. Mortality within 1 year.
3. Other serious adverse events (SAE) within 1 year.

#### Long-term efficacy outcomes

1. Restenosis at 3 years.
2. Symptomatic restenosis at 3 years.
3. Recurrent ischaemic events (stroke or TIA) beyond 30 days through 3 years.
4. mRS Score at 3 years.

### Sample size

We assume the primary efficacy outcome (restenosis at 6 months) at 20% in the BMS stenting group^16 17^ and 15% in the DCB angioplasty group,^12 18 19^ with a target difference of 5%. A likelihood score method for non-inferiority with a margin of 12% and α=0.025 (one sided) shows that 79 patients per treatment group will provide a power of 80%. Since it is difficult to perform DSA at 6-month follow-up, we assume 20% of patients will drop out, and totally 198 patients (99 per treatment group) are needed.

### Statistical analyses

In this trial, we have predefined the switching between non-inferiority and superiority tests for the primary efficacy outcome.^20^ Precisely, a non-inferiority test is first conducted. If the non-inferiority hypothesis is established, a superiority test is further carried out. In case the restenosis rate at 6 months in the DCB group is significantly lower than that in the BMS group (two-sided p<0.05), it is considered that the superiority hypothesis is established (ie, the superiority cut-off value is set to zero) and the study conclusion is superiority; otherwise, the study conclusion is non-inferiority. When the non-inferiority hypothesis is not established, the study conclusion does not support non-inferiority or superiority.

All primary and secondary outcomes are assessed in the intention-to-treat set (cases randomised and treated with study devices) as the main analysis. Continuous variates (median and interquartile range [IQR]) are compared by two-tailed independent-sample Wilcoxon test. Categorical variates are compared by the likelihood ratio χ^2^ test or the Fisher’s exact test. Risk differences (RD) between the two groups with 95% confidence intervals (CI) are determined by the normal approximation or Newcombe method. We examine the rates of restenosis and symptomatic restenosis at 6 months, technical success and procedure-related complications by the generalised linear model with risk ratios (RR) and 95% CIs presented. The probabilities of stroke or death within 30 days, recurrent ischaemic events beyond 30 days through 1 year, mortality and other SAE within 1 year are assessed by the Cox proportional hazard regression model, and hazard ratios (HR) with 95% CIs are calculated. An ordinal logistic regression model is performed to assess the effect of a shift of mRS at 1 year towards a better functional outcome, with odds ratios (OR) and 95% CIs presented.

All primary and secondary outcomes are assessed in the per-protocol set (cases treated based on randomisation, excluding crossovers, with no major protocol deviation) as a sensitivity analysis. An additional sensitivity analysis of all primary and secondary outcomes is also carried out following multiple imputation of missing data. The widths of the CIs for the examined secondary outcomes are not adjusted for multiple comparisons and may not be used for hypothesis testing. Two-sided p<0.05 indicates statistical significance. SAS V.9.4 is used for data analysis.

### Study organisation

The steering committee holds meetings two times a year for overseeing the study and providing strategic direction. The investigators in the main study centre will meet regularly with the team performing the trial at the contract research organisation, reviewing the trial progress and data quality at least monthly. The data safety and monitoring board (DSMB) of this study consists of an independent statistician and academicians and will have meetings at regular intervals for reviewing the study progress to ensure the study adheres to the ethical standards as well as patient safety. The DSMB will apply the predefined protocol definitions and review all available aggregated data to adjudicate the occurrence of safety outcomes and other adverse events, in order to provide counsel to the sponsor on the safety of patients already enrolled and to be enrolled, as well as monitor the ongoing validity and scientific integrity of the trial. CEC and Core-Lab members reviewing clinical and imaging findings, respectively, will be blinded for treatment group.

## Discussion

ICAD has high prevalence, likely representing the the most common aetiology of stroke globally. Even following BMT, stroke/TIA recurrence is high in sICAD cases, particularly in individuals with high-grade stenosis and haemodynamic disorders, indicating further therapeutic options are required. To date, BMS stenting is applied for second-line treatment in sICAD. However, restenosis still has an elevated rate, representing an important issue for the latter procedure. Recently, growing experience tells us that DCB and DES are superior to the conventional BMS in the treatment of sICAD. Jia *et al* reported a multicentre RCT suggesting that DES were superior to BMS for symptomatic high-grade intracranial stenosis with a low risk of in-stent restenosis (10% vs 32%, p<0.001) and ischaemic stroke recurrence (1% vs 9%, p=0.03).^9^ DCB has further advantages compared with DES. In the tortuous neurovascular anatomy, DCB is more flexible due to a softer distal tip compared with DES, thus enabling the operator to reach more distant lesions. DCB does not leave residual foreign bodies, thus exerting a positive impact on the possibility of subsequent adverse material-tissue reactions and local flow dynamics. In contrast to DES, DCB offers a uniform antiproliferative drug coverage of the diseased vessel lumen. Furthermore, a shorter duration of recommended DAPT might be reasonable for DCB given the lower risk of delayed endothelialisation and subsequent thrombosis when compared with DES.^21^ Correspondingly, DCB angioplasty may represent a promising alternative to BMS or DES stenting for the treatment of patients with sICAD. However, severe arterial dissection and elastic recoil remain major issues after using DCB in sICAD. Current data from several small studies indicate DCB angioplasty is safe and feasible in sICAD cases with high-grade stenosis.^10 22^ Thus, larger randomised trials are warranted to confirm the efficacy and safety of this procedure.

DR. BEYOND is a phase III, randomised controlled study aiming to assess the efficacy and safety of Taijieweiye intracranial paclitaxel-coated balloon angioplasty in sICAD cases with high-grade stenosis (≥70%–99%) and haemodynamic disorders (symptoms resulting from hypoperfusion with poor collaterals). The primary efficacy and safety outcomes of this study are restenosis at 6 months and stroke/death within 30 days, respectively. DR. BEYOND enrolled the first patient on 16 July 2021. As of 29 March 2023, all participants were enrolled. The study will be completed, including the collection of 1-year and 3-year outcomes, by late June 2026.

In the proposed trial, certain shortcomings of BMS will be addressed. The present manuscript outlines the rationale and design of DR. BEYOND study. We estimate that this trial will allow for a critical reappraisal of the role of intracranial angioplasty for selected sICAD patients with high-grade stenosis. Further investigation into the efficacy and safety of DCB is warranted, such as comparing DCB with DES or BMT.

## Conclusions

DR. BEYOND will provide objective data to determine whether DCB angioplasty is non-inferior or even superior to BMS stenting in sICAD cases, which might propose an alternative option for addressing sICAD.

## Supplementary material

10.1136/svn-2024-003259 online supplemental file 1

## Data Availability

Data are available on reasonable request. No data are available.
