# Supplementary material for: DRug-coated Balloon for Endovascular treatment of sYmptOmatic intracraNial stenotic Disease (DR. BEYOND): the protocol of a multicentre randomised trial
Source: Stroke Vasc Neurol. 2024 Jul 23;10(2):e003259. doi: 10.1136/svn-2024-003259 (PMC12107454; doi:10.1136/svn-2024-003259)
Supplement: online supplemental file 1 [file svn-10-2-s001.docx]

**Supplemental Material**

**Table S1.** Assessment and follow-up schedule

| **Time point** |  | **Enrollment and Treatment** | | |  | **Follow-up** | | | | | | | | |
| --- | --- | --- | --- | --- | --- | --- | --- | --- | --- | --- | --- | --- | --- | --- |
|  |  | **Pre-procedural screening** |  | **During the procedure** |  | **3 days or discharge** |  | **30 days** |  | **6 months** |  | **1 year** |  | **3 years** |
| **Window phase** |  | **-14 to 0 day** |  | **0 day** |  | **whichever comes first** |  | **±3 days** |  | **±14 days** |  | **±1 month** |  | **±3 months** |
| Informed consent |  | X |  |  |  |  |  |  |  |  |  |  |  |  |
| Inclusion/exclusion criteria |  | X |  | X |  |  |  |  |  |  |  |  |  |  |
| Past history/demographic data |  | X |  |  |  |  |  |  |  |  |  |  |  |  |
| Vital signs |  | X |  |  |  | X |  |  |  |  |  |  |  |  |
| Blood routine |  | X |  |  |  | X |  |  |  |  |  |  |  |  |
| Blood biochemistry |  | X |  |  |  | X |  |  |  |  |  |  |  |  |
| Coagulation function |  | X |  |  |  | X |  |  |  |  |  |  |  |  |
| Clopidogrel resistance test^*^ |  | X |  |  |  |  |  |  |  |  |  |  |  |  |
| ECG |  | X |  |  |  |  |  |  |  |  |  |  |  |  |
| Chest X-ray |  | X |  |  |  |  |  |  |  |  |  |  |  |  |
| CT+CTA or MRI+MRA^†^ |  | X |  |  |  |  |  |  |  |  |  |  |  |  |
| DSA |  |  |  | X |  |  |  |  |  | X |  |  |  | X |
| NIHSS score |  | X |  |  |  | X |  |  |  |  |  |  |  |  |
| mRS score |  | X |  |  |  | X |  | X |  | X |  | X |  | X |
| Use of endovascular devices |  |  |  | X |  |  |  |  |  |  |  |  |  |  |
| Concomitant medication |  | X |  | X |  | X |  | X |  | X |  | X |  | X |
| AE/SAE |  |  |  | X |  | X |  | X |  | X |  | X |  | X |
| Defects of study devices |  |  |  | X |  |  |  |  |  |  |  |  |  |  |

^*^It is recommended to perform thromboelastography and/or CYP2C19 genotype test to detect the presence of clopidogrel resistance, and clopidogrel is shifted to ticagrelor in patients with clopidogrel resistance.

^†^It is recommended to perform CTP or PWI examination to confirm the presence of hypoperfusion in the territory distal to the target lesion.

Abbreviations: AE, adverse event; CTA, CT angiography; CTP, CT perfusion; DSA, digital subtraction angiography; ECG, electrocardiogram; MRA, MR angiography; mRS, modified Rankin Scale; NIHSS, National Institutes of Health Stroke Scale; PWI, perfusion weighted imaging; SAE, serious adverse event
